# Supplementary figures and images for: Dynamic Functional Connectivity of Emotion Processing in Beta Band with Naturalistic Emotion Stimuli
Source: Brain Sci. 2022 Aug 19;12(8):1106. doi: 10.3390/brainsci12081106 (PMC9405988; doi:10.3390/brainsci12081106)

## Group-01

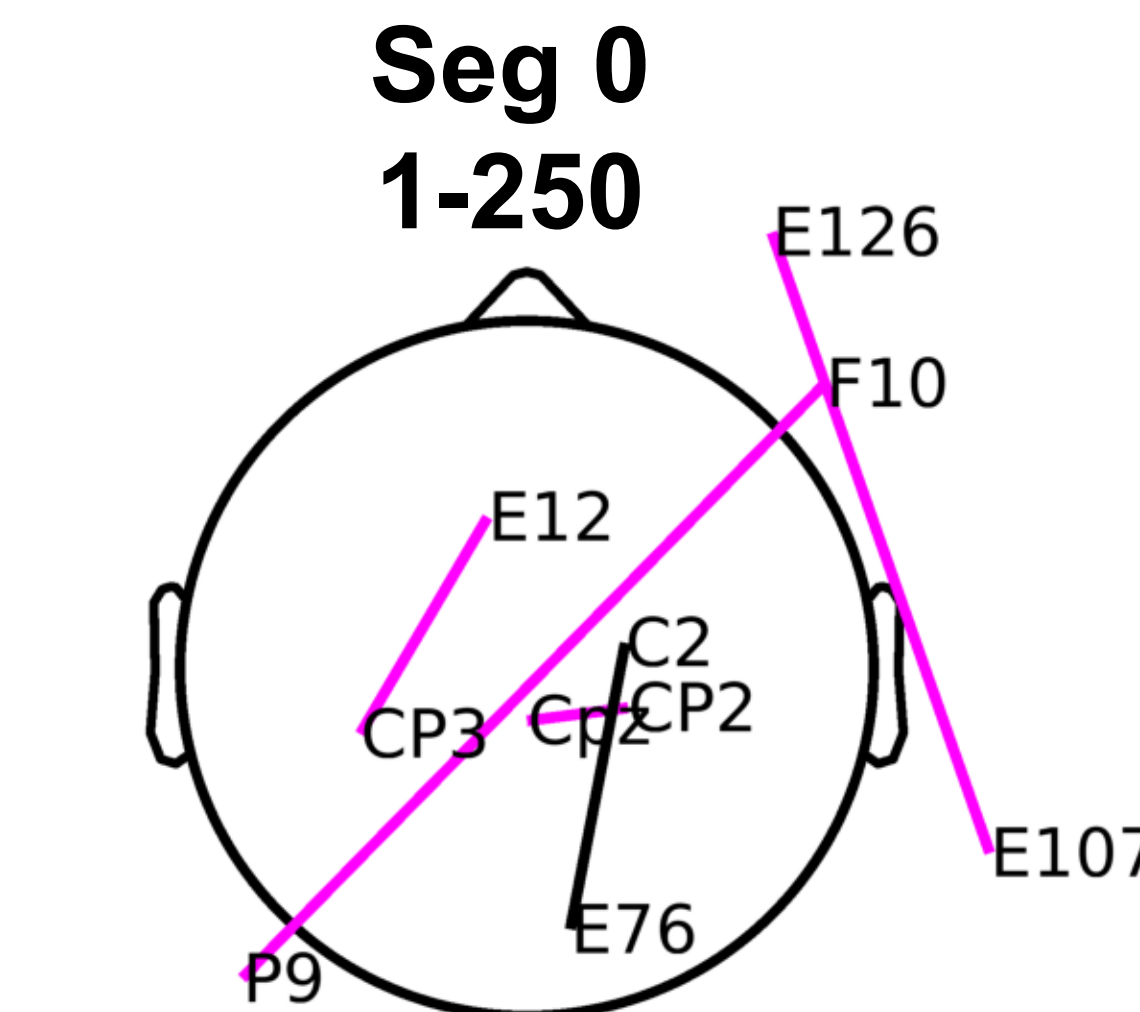

## Group-02

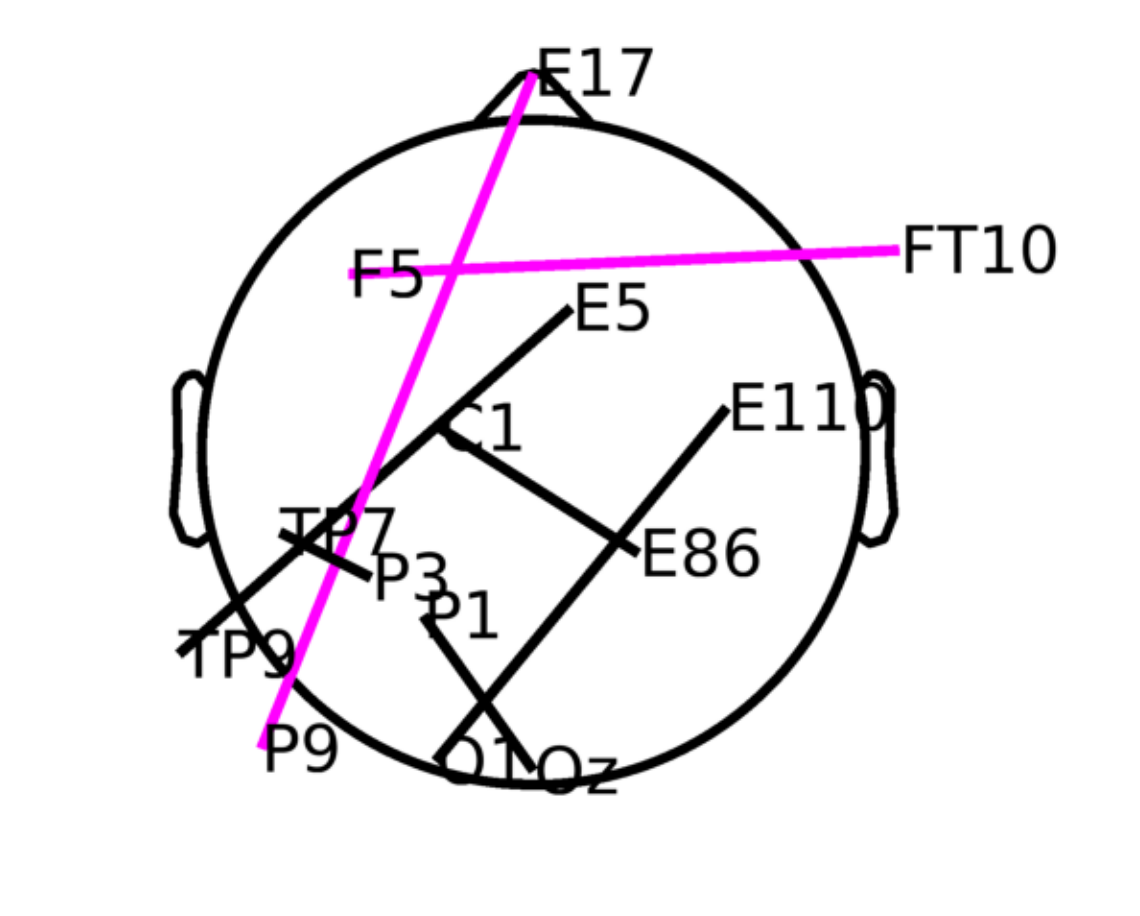

## Group-03

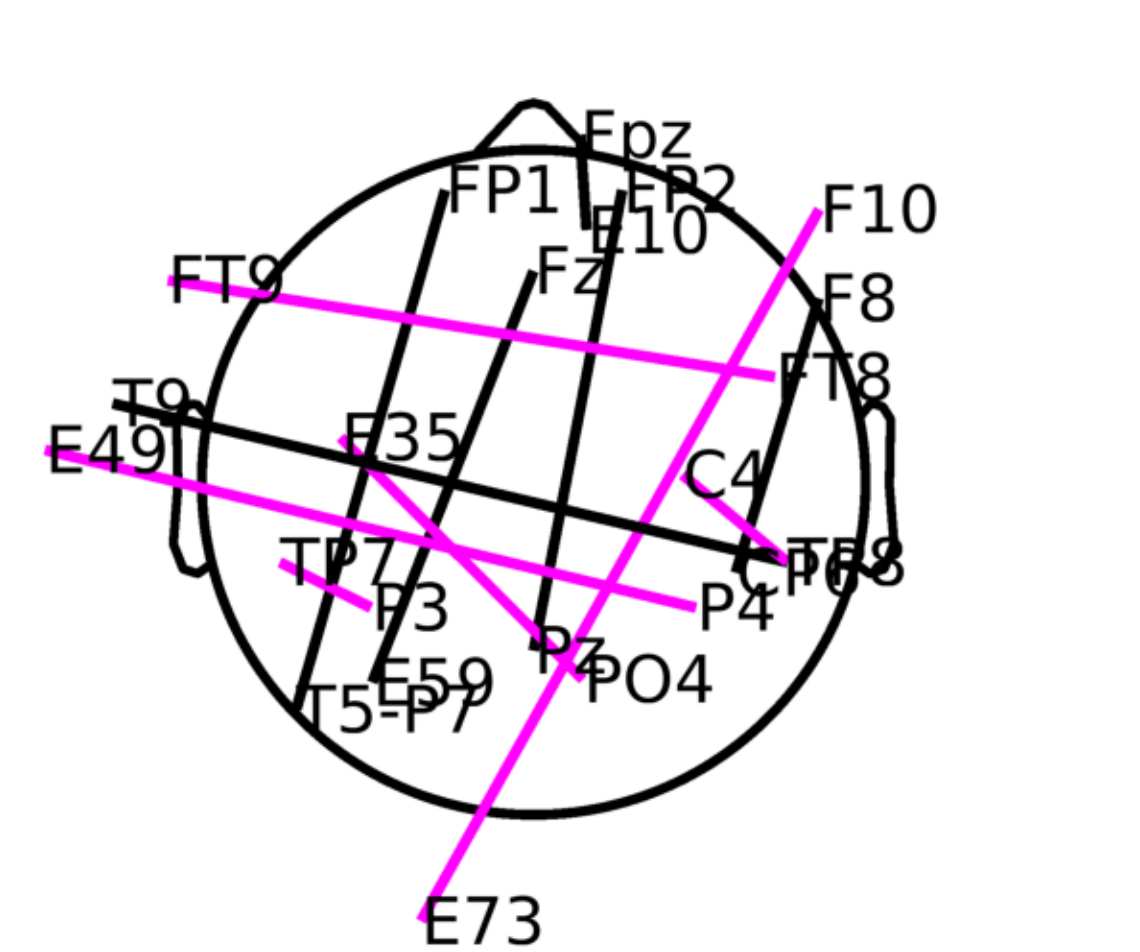

## Group-04

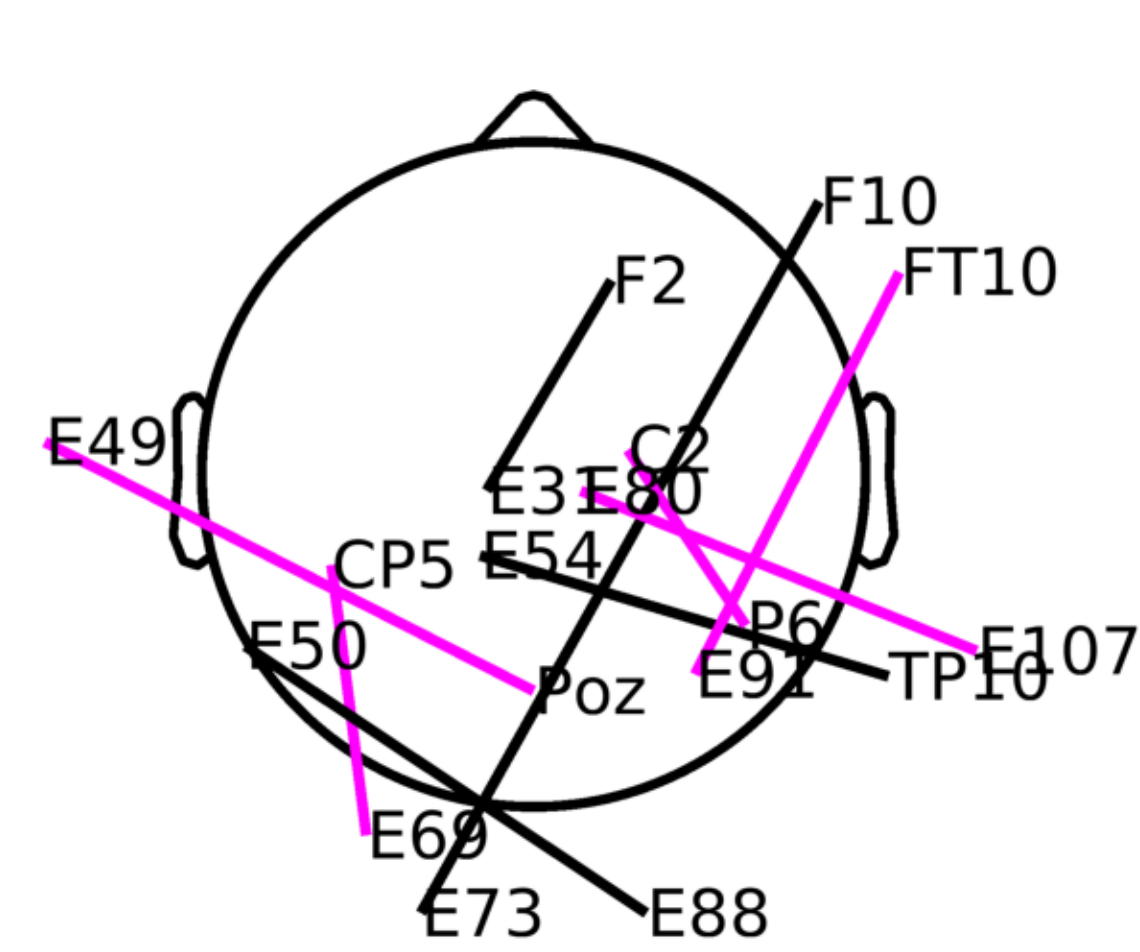

roup-05

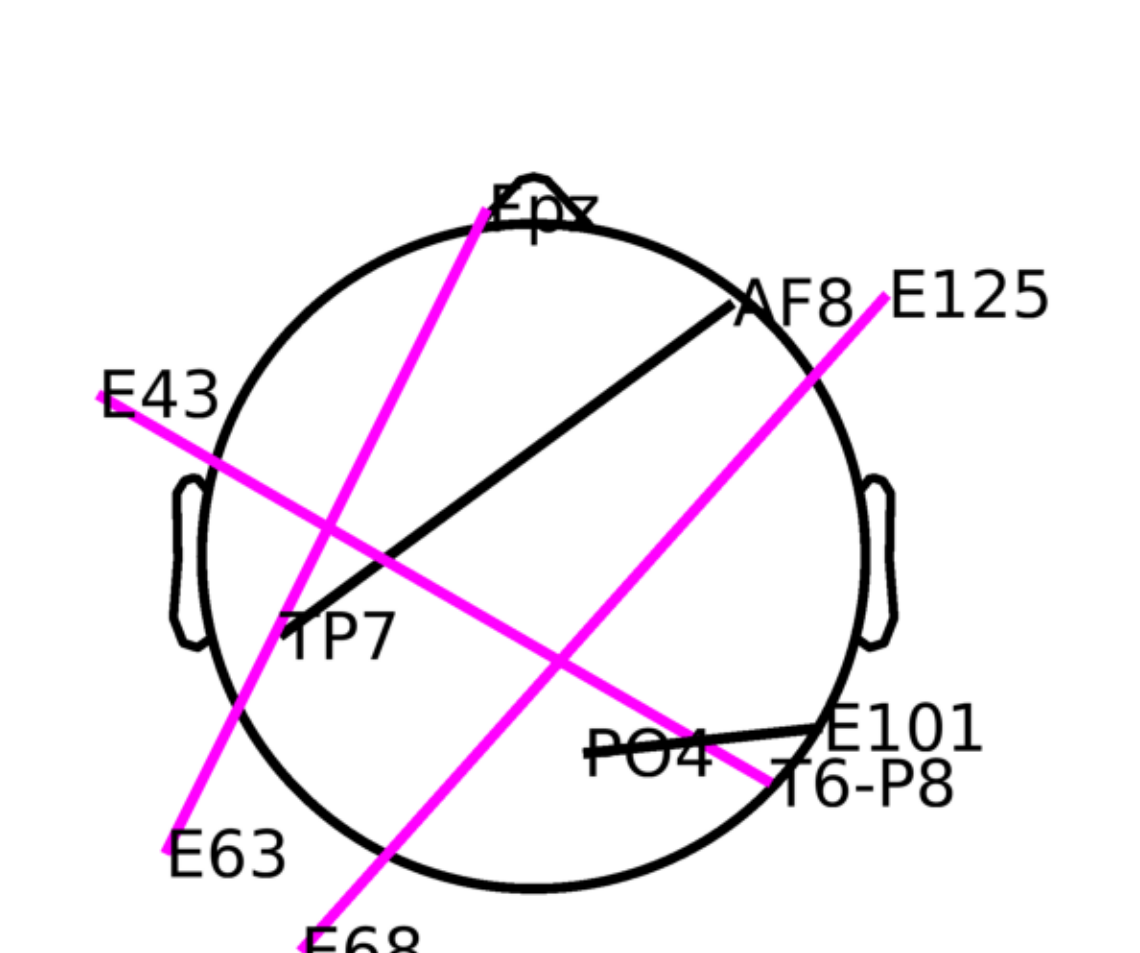

90-dnc

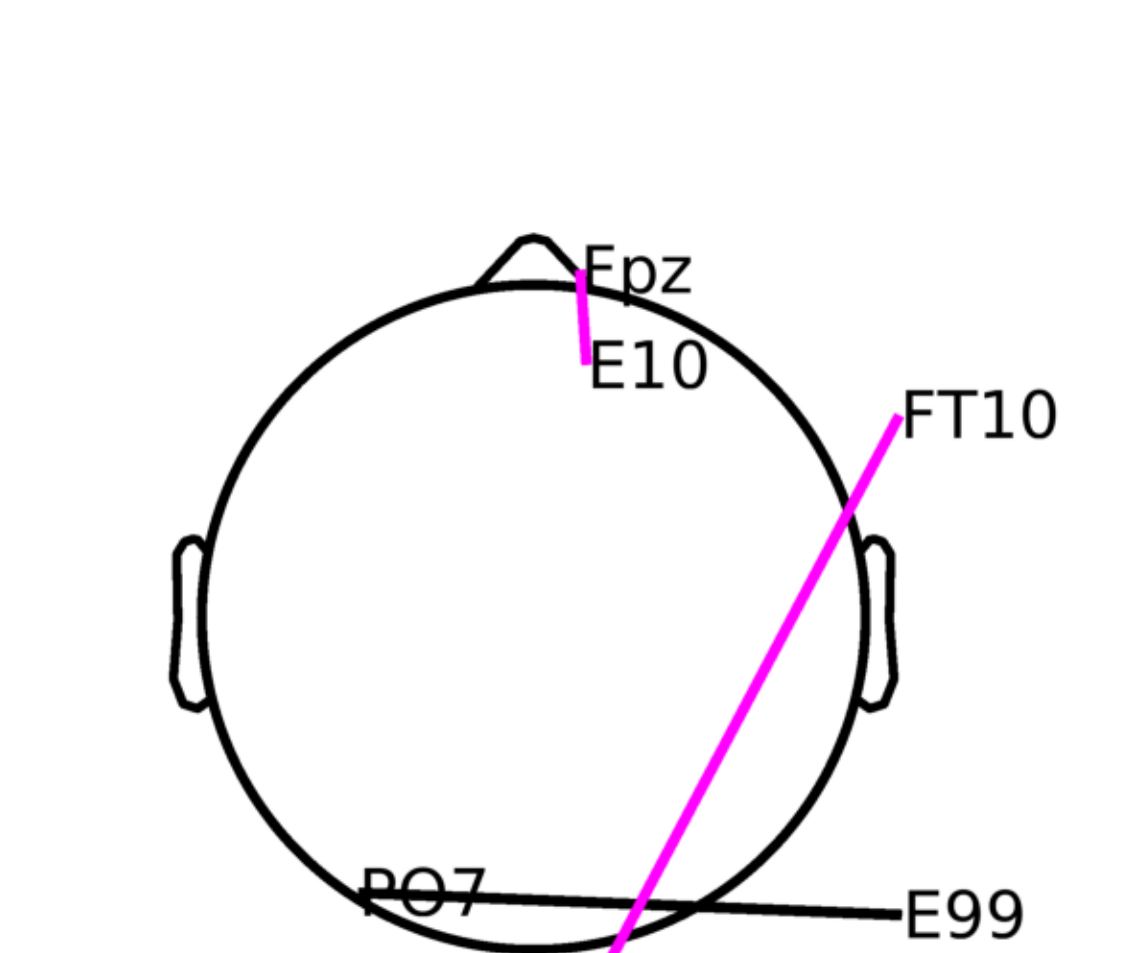

20-dno

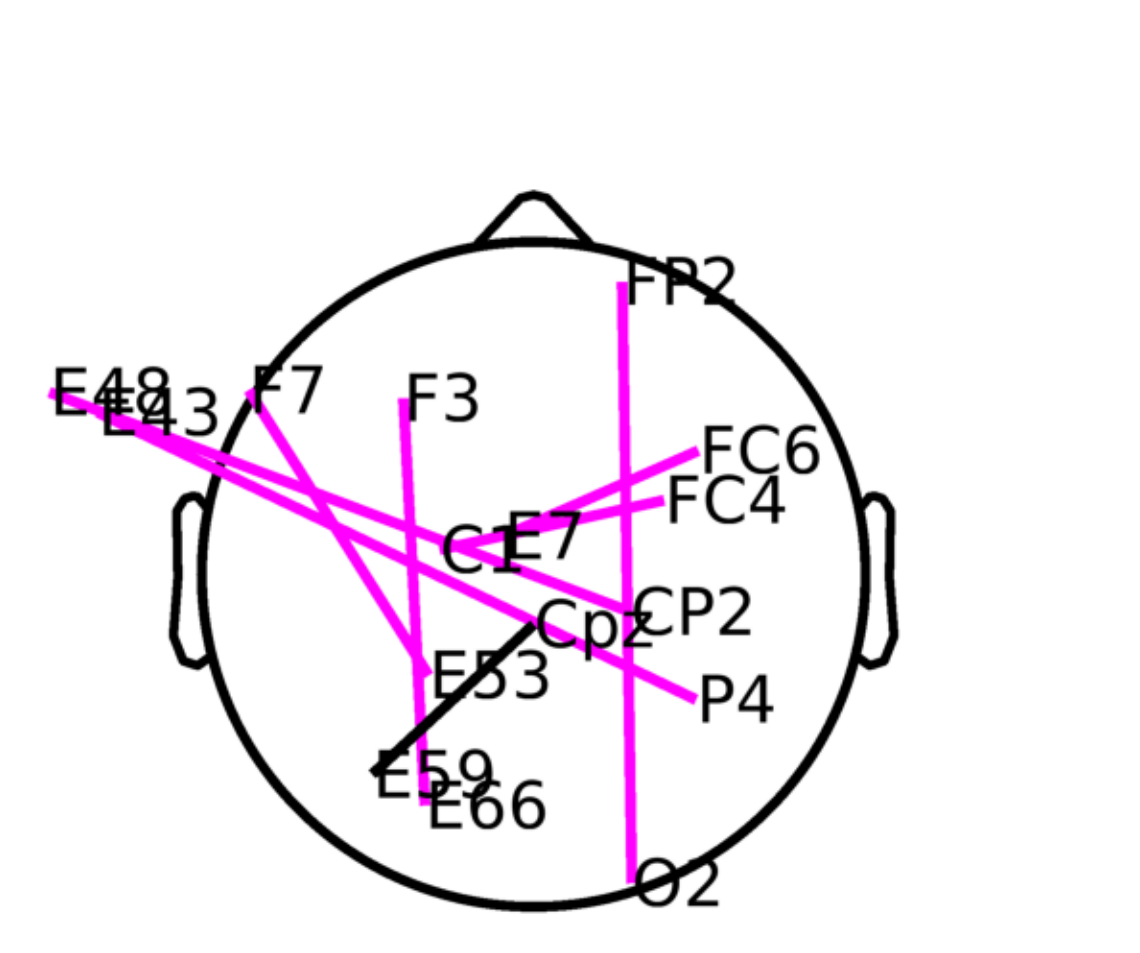

**roup-08**

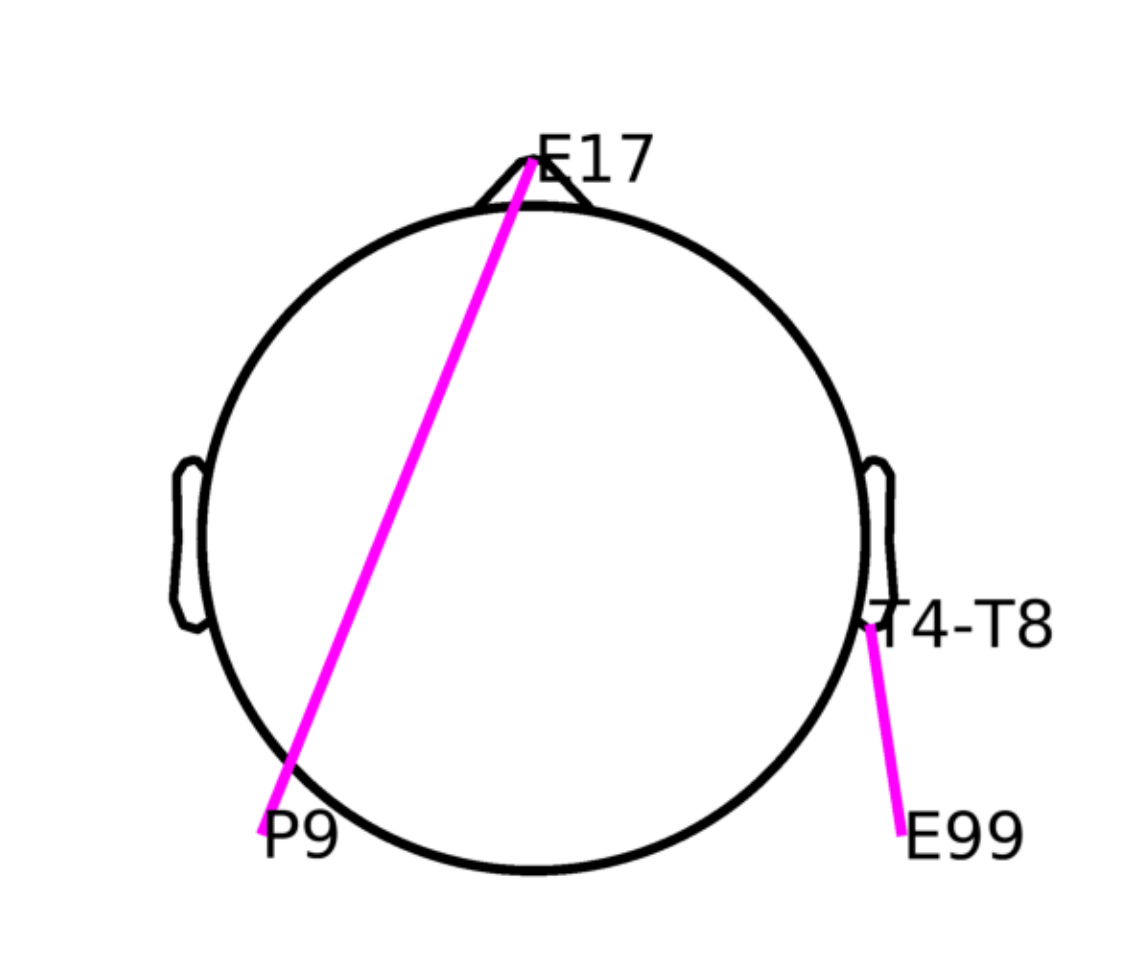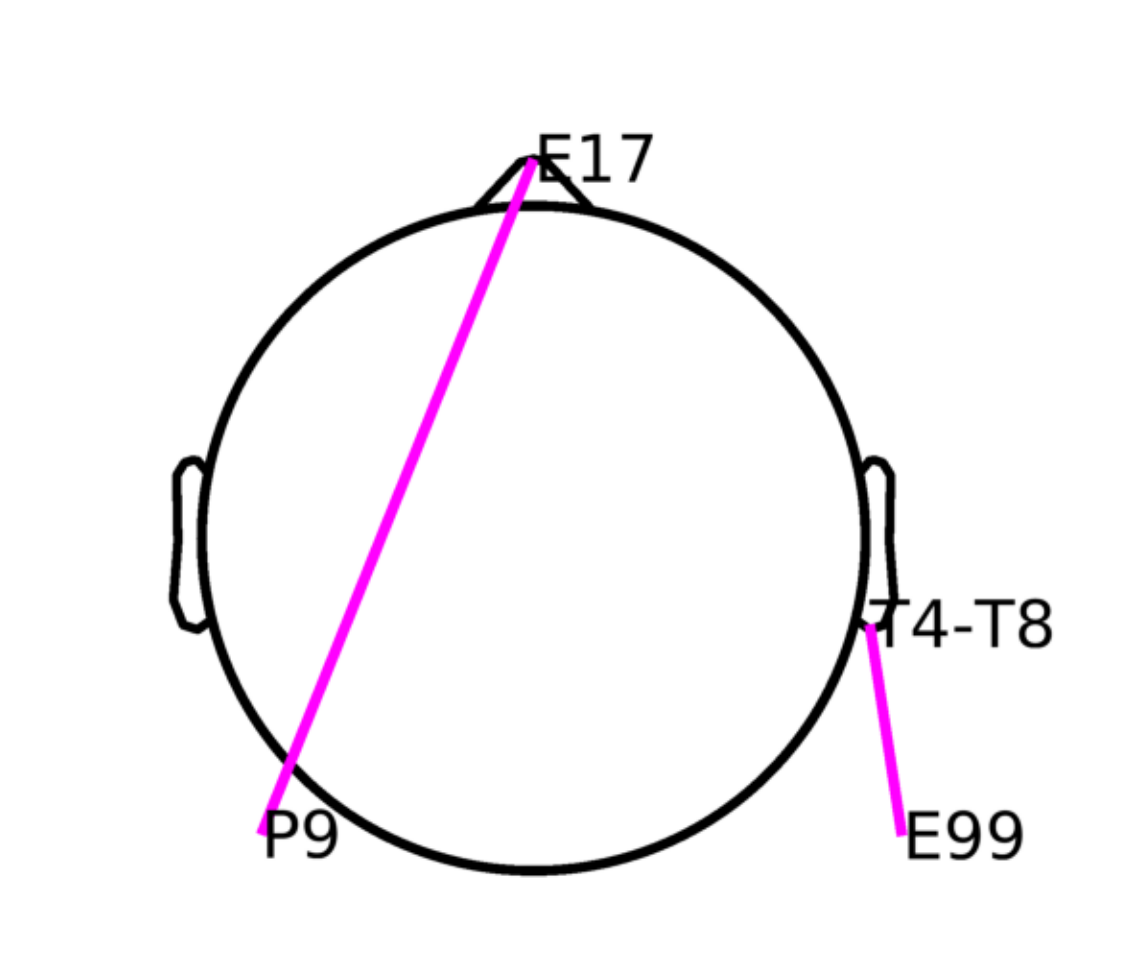

Supplement: Supplementary file 1 [file brainsci-12-01106-s001.zip › Figure S3-1.pdf]

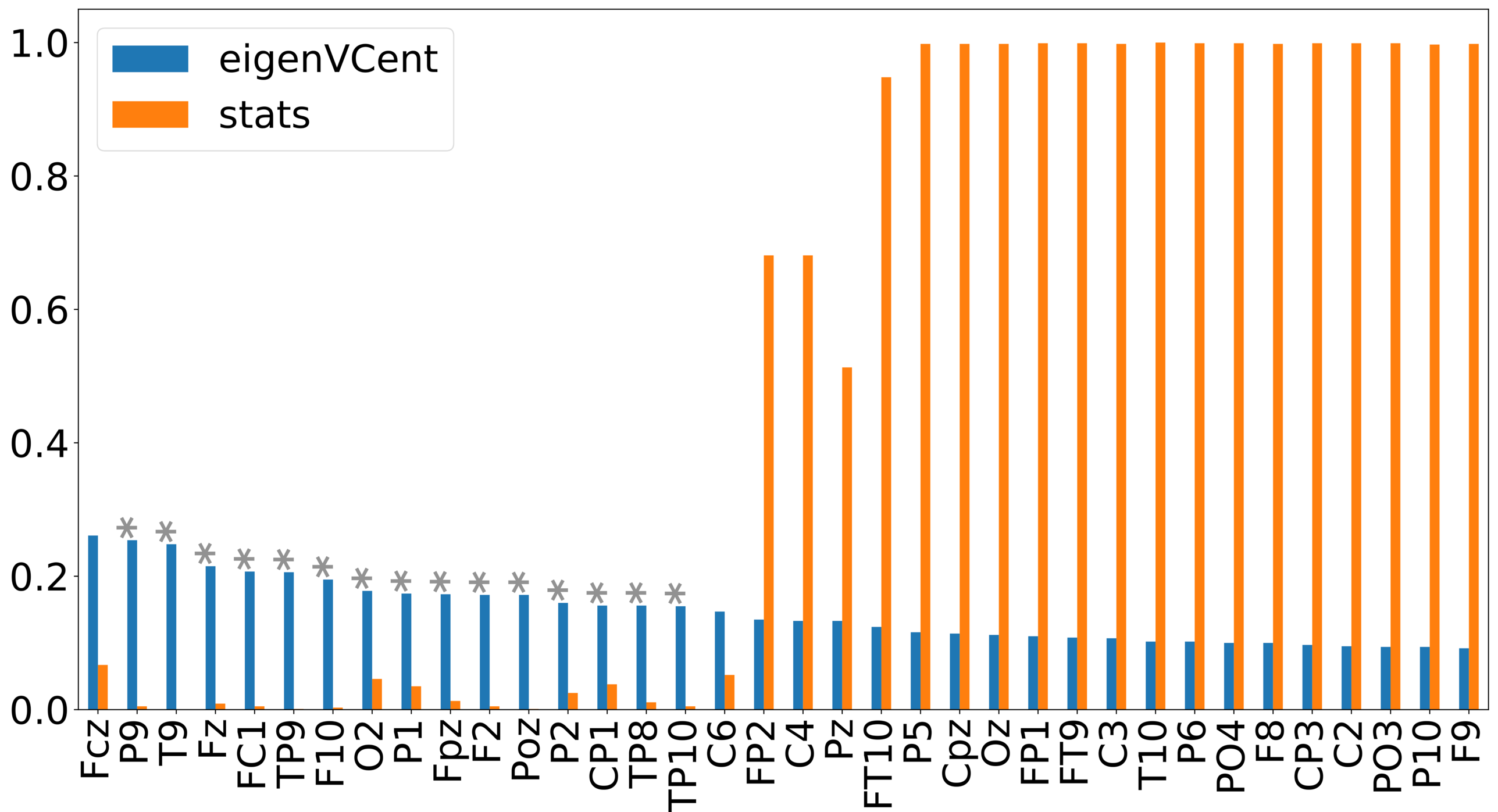

(a)

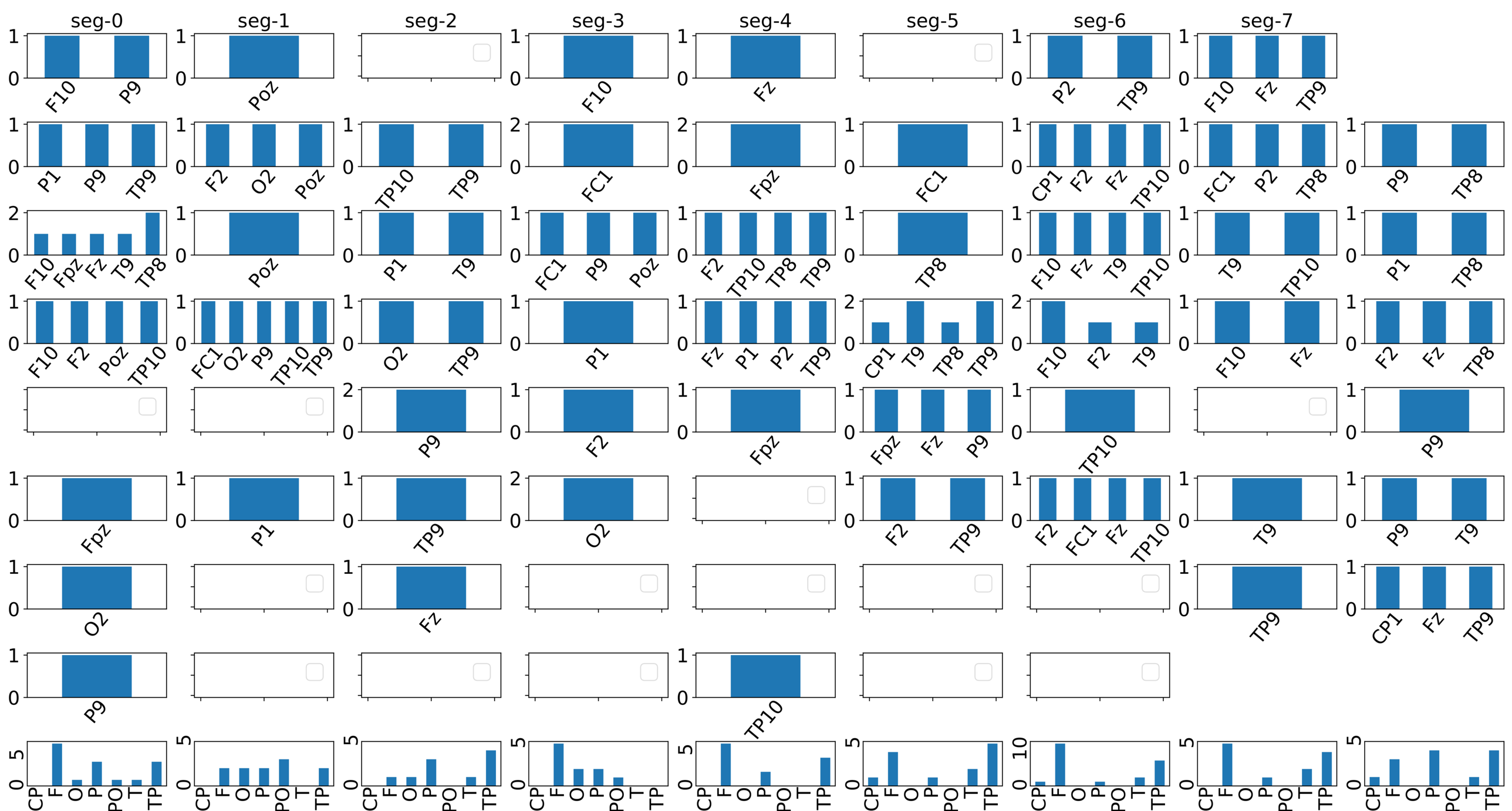

(b)

Supplement: Supplementary file 1 [file brainsci-12-01106-s001.zip › Figure S5-1.pdf]

seg-0

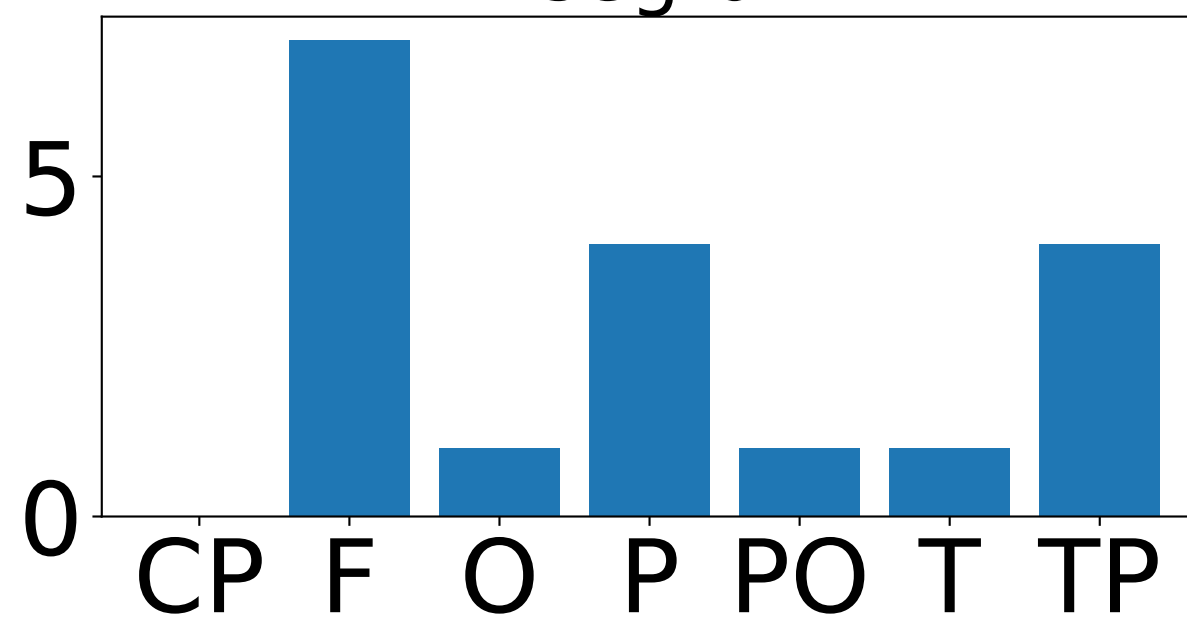

seg-1

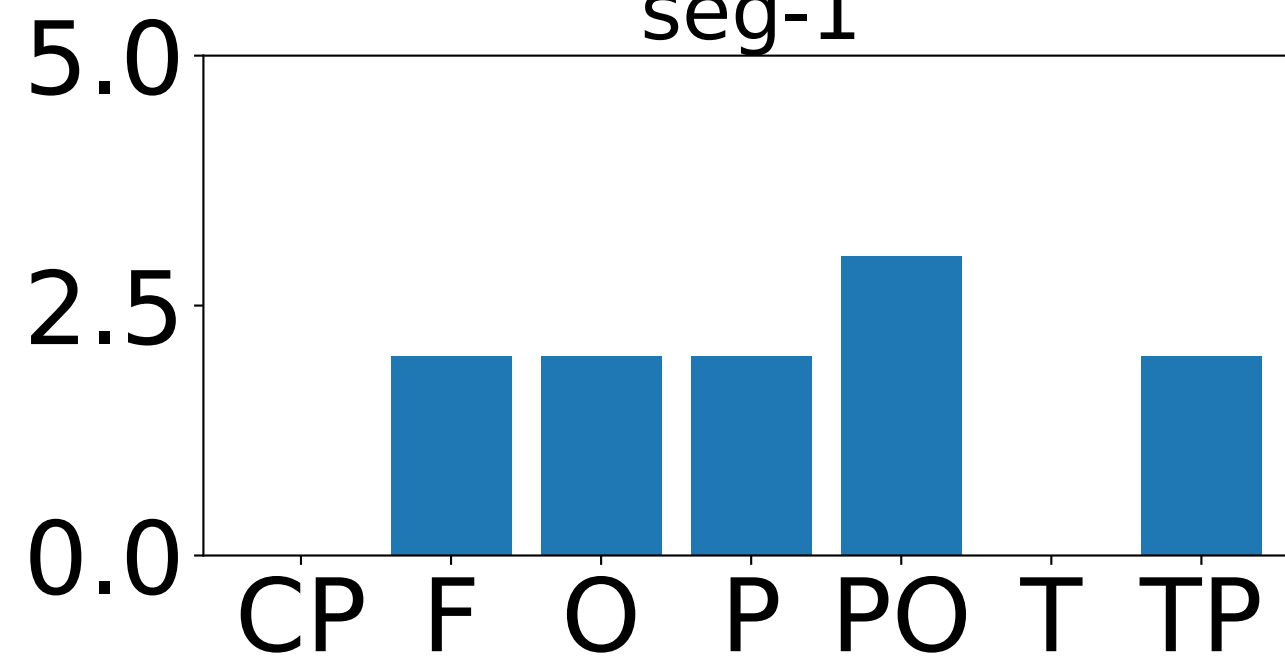

seg-2

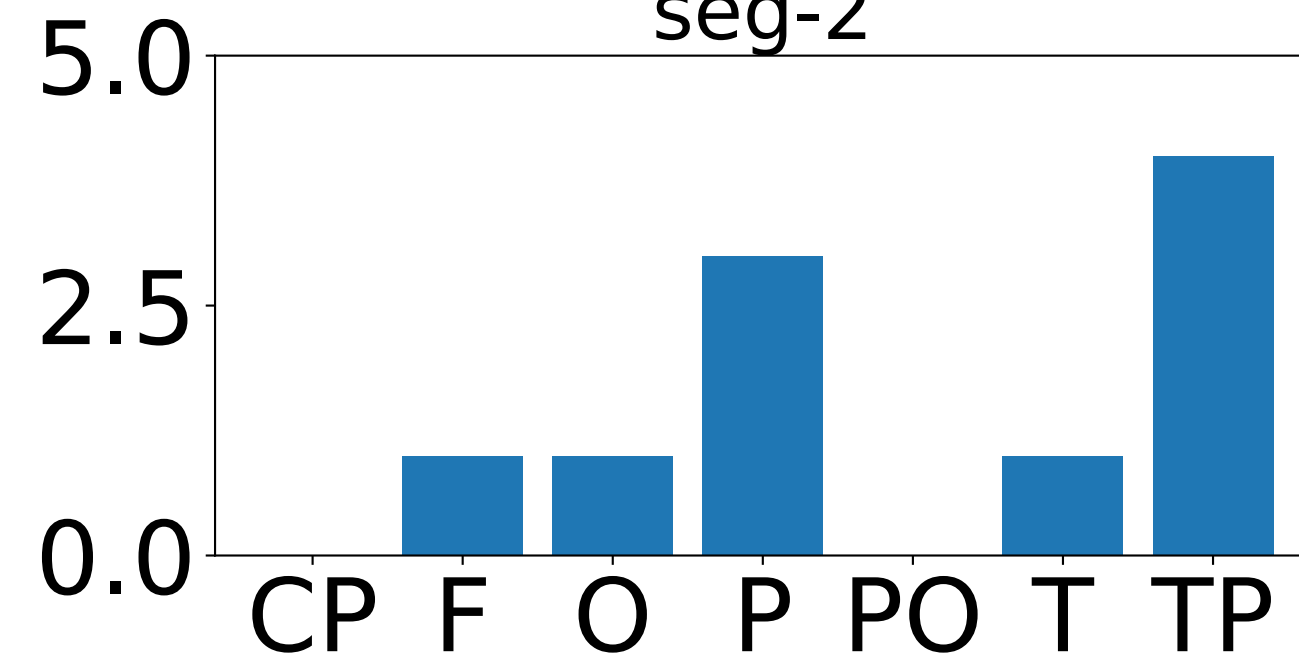

seg-3

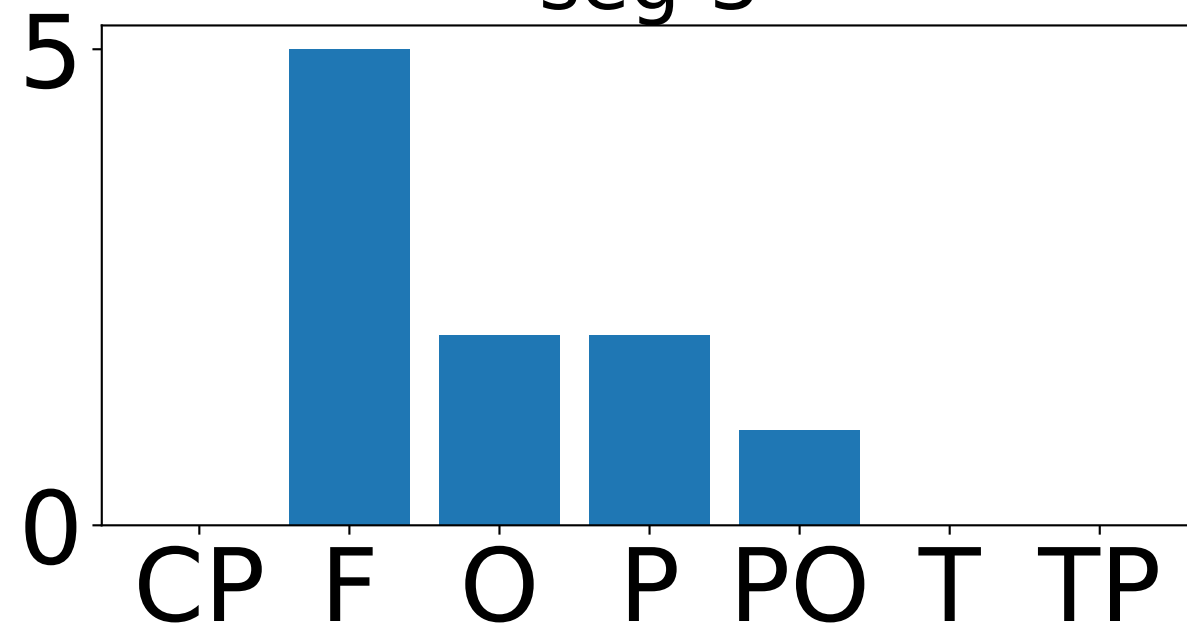

seg-4

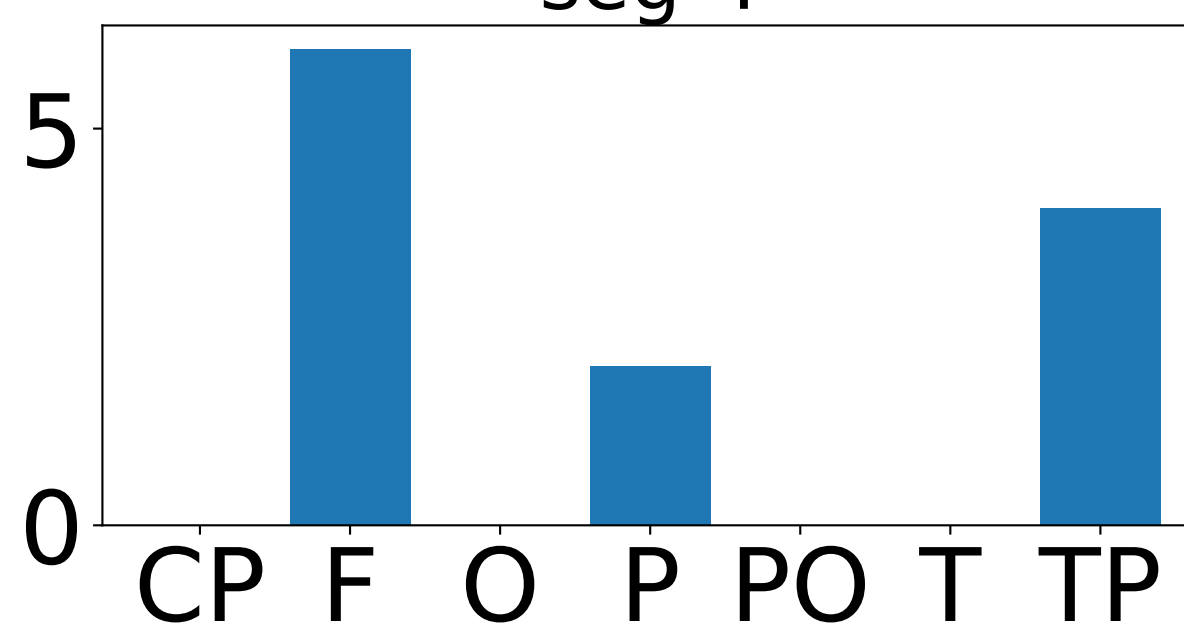

seg-5

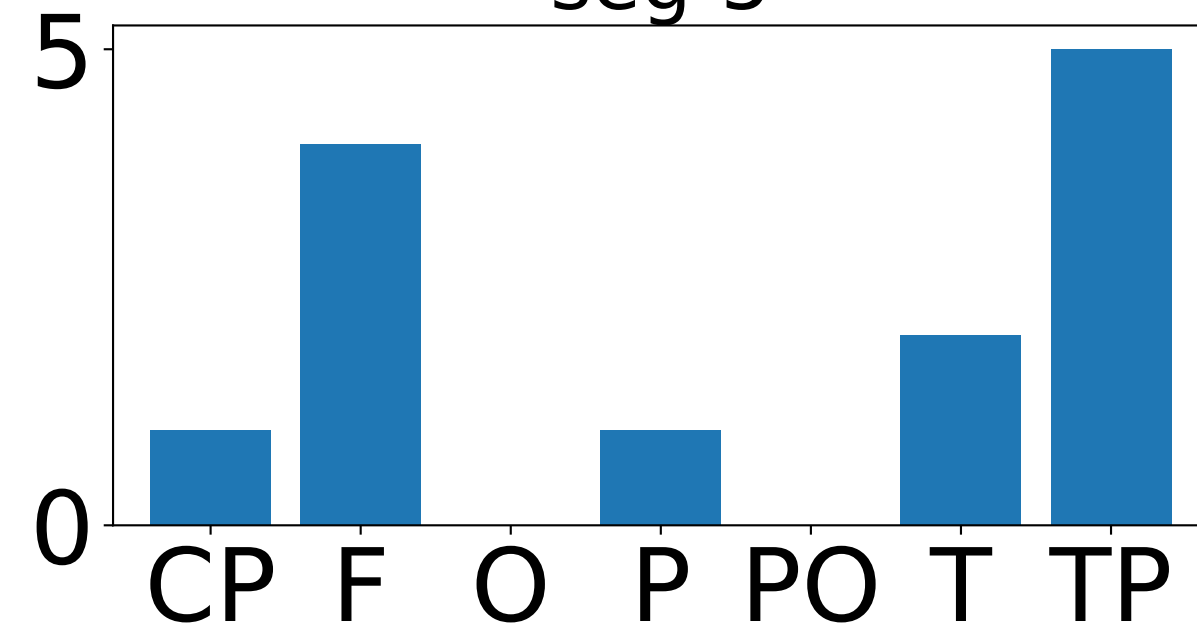

seg-6

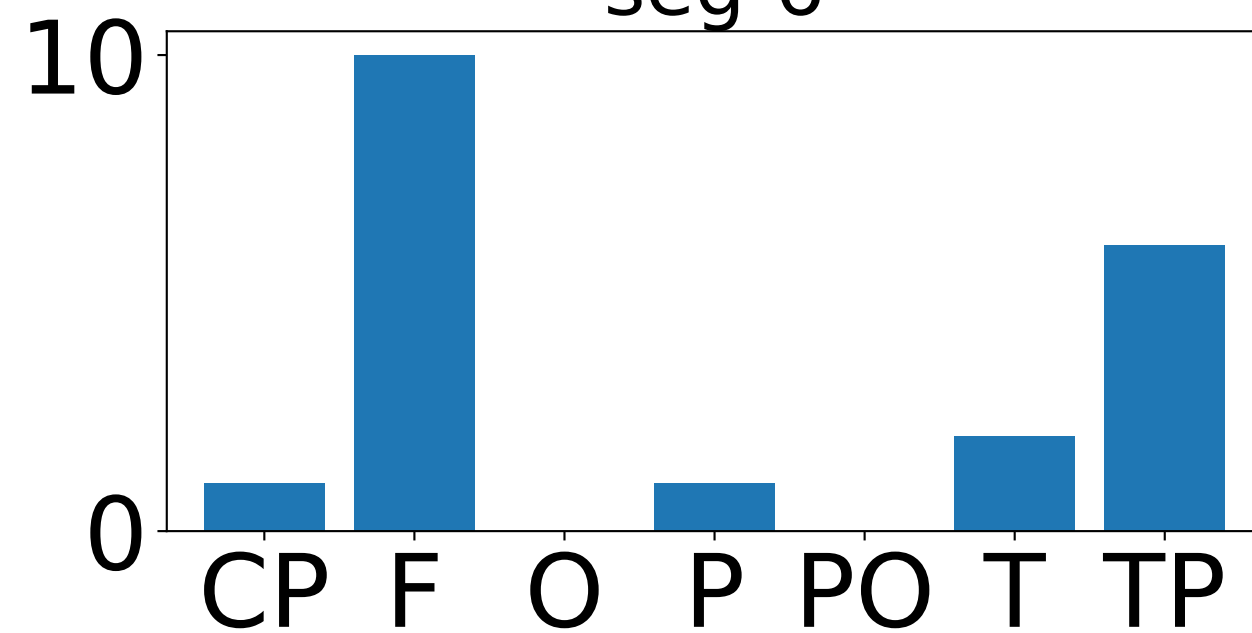

seg-7

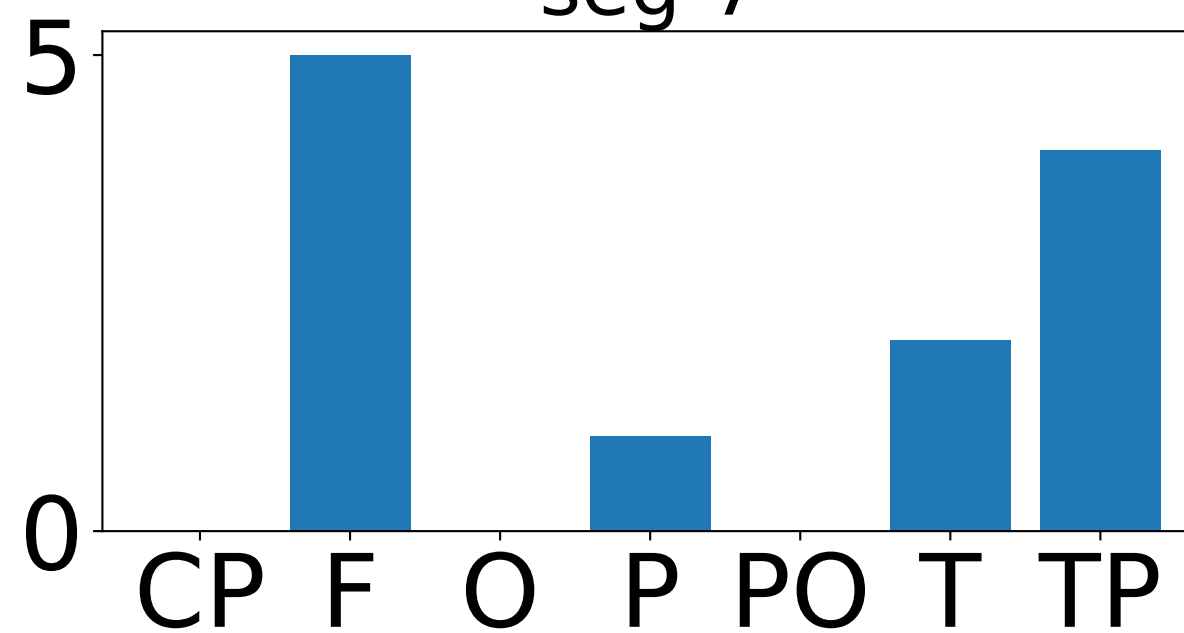

seg-8

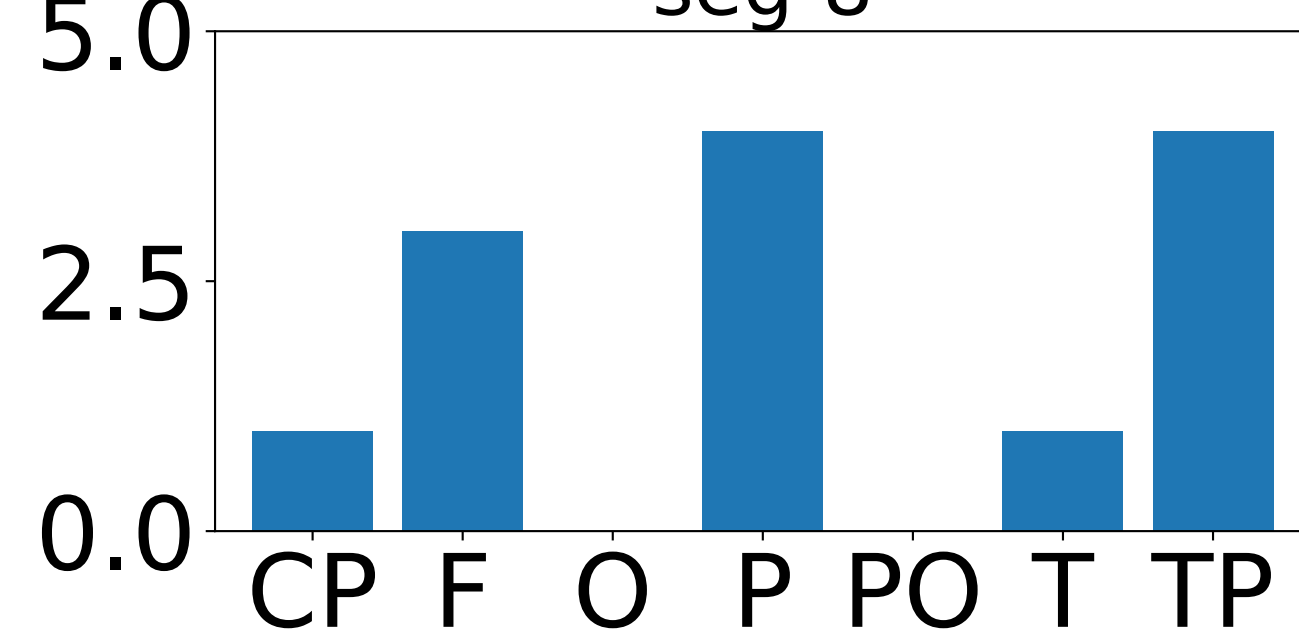

Supplement: Supplementary file 1 [file brainsci-12-01106-s001.zip › Figure S5-2.pdf]
